# Identification of a Novel *De Novo* Mutation Associated with *PRKAG2* Cardiac Syndrome and Early Onset of Heart Failure

**DOI:** 10.1371/journal.pone.0064603

**Published:** 2013-05-31

**Authors:** Yang Liu, Rong Bai, Lin Wang, Cuntai Zhang, Ruifu Zhao, Deli Wan, Xinshan Chen, Gabriel Caceres, Daniel Barr, Hector Barajas-Martinez, Charles Antzelevitch, Dan Hu

**Affiliations:** 1 Department of Cardiology, Tongji Hospital, Tongji Medical College, Huazhong University of Science and Technology, Wuhan, China; 2 Cardiovascular Department, Guangdong Cardiovascular Institute, Guangdong General Hospital, Guangzhou, China; 3 Department of Geriatrics, Tongji Hospital, Tongji Medical College, Huazhong University of Science and Technology, Wuhan, China; 4 Department of Forensic Medicine, Tongji Medical College, Huazhong University of Science and Technology, Wuhan, China; 5 Department of Molecular Genetics and Experimental Cardiology, Masonic Medical Research Laboratory, Utica, New York, United States of America; 6 Department of Chemistry and Biochemistry, Utica College, Utica, New York, United States of America; University of Minnesota, United States of America

## Abstract

**Introduction:**

The major structure elements of the AMP-activated protein kinase (AMPK) are α, β, and γ sunbunits. Mutations in γ2 subunit (*PRKAG2*) have been associated with a cardiac syndrome including inherited ventricular preexcitation, conduction disorder and hypertrophy mimicking hypertrophic cardiomyopathy. The aim of the present study was to identify *PRKAG2* syndrome among patients presenting with left ventricular hypertrophy (LVH).

**Methods and Results:**

Nineteen unrelated subjects with unexplained LVH were clinically and genetically evaluated. Among 4 patients with bradycardia, manifestations of preexcitation were only found in a 19 year old male who also developed congestive heart failure 3 years later. Electrophysiological study of this case identified the coexistence of an AV accessory pathway and AV conduction defect. Histological analysis of his ventricular tissue isolated by biopsy confirmed excessive glycogen accumulation, prominent myofibrillar disarray and interstitial fibrosis. Direct sequencing of his DNA revealed a heterozygous mutation in *PRKAG2* consisting of an A-to-G transition at nucleotide 1453 (c.1453A>G), predicting a substitution of a glutamic acid for lysine at highly-conserved residue 485 (p.Lys485Glu, K485E), which was absent in his unaffected family members and in 215 healthy controls. To assess the role of K485 in the structure and function of the protein, computational modeling calculations and conservation analyses were performed. Electrostatic calculations indicate that K485 forms a salt bridge with the conserved D248 residue in the AMPK β subunit, which is critical for proper regulation of the enzyme, and the K485E mutant disrupts the connection.

**Conclusions:**

Our study identifies a novel *de novo PRKAG2* mutation in a young, in which progression of the disease warrants close medical attention. It also underlines the importance of molecular screening of *PRKAG2* gene in patients with unexplained LVH, ventricular preexcitation, conduction defect, and/or early onset of heart failure.

## Introduction

Unexplained left ventricular hypertrophy (LVH) is a common finding, the majority of which is usually attributed to hypertrophic cardiomyopathy (HCM), an autosomal dominant hereditary disease caused principally by mutations in genes encoding the sarcomeric proteins [1]. However, other disease-causing genes have also been identified in patients with unexplained LVH, such as the *PRKAG2* gene, which encodes the AMP-activated protein kinase (AMPK) γ2 regulatory subunit [2], [3]. Defects in *PRKAG2* are associated with a recently described cardiac syndrome triad consisting of familial ventricular preexcitation (Wolff-Parkinson-White syndrome, WPW), conduction system disease and cardiac hypertrophy mimicking HCM [4]. Histological studies of myocardial tissue from affected individuals [5]–[7] and transgenic mice expressing mutant forms (N488I [8], R302Q [9], R531G [10] and T400N [11], respectively) of the *PRKAG2* gene confirmed glycogen storage as the pathologic basis for this cardiac syndrome. Because of similar echocardiographic features, *PRKAG2* disease could be misdiagnosed as HCM. Unlike HCM, however, individuals with the *PRKAG2* mutations have a higher incidence of progressive cardiac conduction disease requiring implantation of a pacemaker [12], [13]. It is therefore important to distinguish hypertrophy associated with *PRKAG2* mutations from that due to sarcomere protein defects. The objective of the present study was to identify *PRKAG2* cardiac syndrome from a cohort of 19 patients with unexplained LVH. Analysis of the clinical characteristics, ECGs, electrophysiological study, serum biomarkers and histology revealed one candidate with suspected *PRKAG2* syndrome. Genetic analysis confirmed the presence of a novel *de novo PRKAG2* mutation and computational modeling validates that it could lead to severe functional changes.

## Methods

### Clinical Evaluations

Between 2009 and 2011, we screened all patients presentingd with LVH at the Department of Cardiology of Tongji Hospital of the Tongji Medical College. All patients were evaluated on the basis of medical history, physical examination, 12-lead ECG and transthoracic echocardiography. LVH was defined as a thickness of the LV septal wall or the LV free wall of at least 13 mm on echocardiography. Cardiac conduction abnormalities were classified as sinoatrial node dysfunction, atrioventricular (AV) node conduction delay, His bundle or bundle branch block or atrioventricular (AV) node conduction delay, His bundle or bundle branch block, or ventricular preexcitation documented by short PR interval (<120 ms), a widened QRS complex (>110 ms) and a delta wave preceding the QRS. Exclusion criteria included 1) LVH secondary to other cardiovascular conditions including aorta or aortic valve disease; 2) isolated left ventricular (LV) apical hypertrophy shown in a 4-chamber echocardiographic view; 3) patients over 50 years of age in whom the cardiac conduction system abnormality attributable to other pathologies such as ischemic disease. The study was carried out in accordance with institutional guidelines for human research and approved by the ethics committee of the hospital. Informed and written consent was obtained from study subjects. All values are reported as Mean ± SEM.

### Echocardiography

All echocardiographic studies were conducted with a Vivid 7 Doppler ultrasonography system (GE Healthcare, Pittsburgh, PA, USA), equipped with a 1.7/3.4 MHz M3S transducers. The images were acquired with the subjects at rest and lying in the lateral decubitus position. Standard echocardiographic studies consisted of M-mode, cross-sectional, and Doppler blood flow measurements as previously described [14]. Cross-sectional image were recorded from the parasternal long-axis view to measure end-systolic diameter of left atrium, and end-diastolic diameter of LV, septal thickness and posterior wall thickness in end-diastole. M-mode tracings from the parasternal long-axis view were used to measure LV ejection fraction.

### Endomyocardial Biopsy and Histological Examination

Specimens of myocardium were biopsied from the right-sided interventricular septum and fixed in formalin and paraffin embedded before they were sectioned. Sections were evaluated after staining with hematoxylin and eosin and Masson’s trichrome using routine protocols. In addition, several specimens were fixed in glutaraldehyde for transmission electron microscopy (TecnaiG^2^12, FEI, Holland), as previously described [15].

### Molecular Genetic Analysis

Genomic DNA was extracted from peripheral blood lymphocytes using a commercial kit (Gentra System, Puregene, Valencia, CA, USA). All exons and intron borders of the *PRKAG2* gene were amplified and directly sequenced from both directions using an ABI PRISM 3100-Avant Automatic DNA Sequencer (Applied Biosystems, Foster City, CA, USA). The sequences of the primers used for *PRKAG2* are shown in Table 1 (RefSeq NM_016203.3). Genomic DNA from 215 healthy individuals was used as control.

**Table 1:** Sequences of primers for *PRKAG2* gene.

Exon	Sense	Antisense
1	5'-CGA GGG TTC CGT AGG AAA G-3'	5'-CTC TAC CCT TTC CCC AAG-3'
2	5'-GAA GGA TAA GGT CTC AGG AAG-3'	5'-GAG GCT CTC TAG TGG GAT AG-3'
3	5'-GCT ACC TCT GTG GAA GAG C-3'	5'-CAC CTG GCA GCT TCG GTG-3'
4	5'-CAC CAG GAA TGG TGA GAG C-3'	5'-CTC ACC ATT TCT CCC TCA GC-3'
5	5'-GTC GCA GCT CAT GCT GAT-3'	5'-CGG CGA GTA AGG ACA AAA GG-3'
6	5'-CCT TGA TCA TAC TGG GAA AAC-3'	5'-CCC GAT AGT GCA AAG GAC TC-3'
7	5'-CCT GGG CAA CAG AGT GAA AC-3'	5'-CCC TGC CAG CAA GAA TGT TC-3'
8	5'-CTC TGC TTG ATA TCT TCG AAG-3'	5'-CAC CAT CAG CAC ACC ATA CC-3'
9	5'-GTG ATC TGC CCA CCT TGA TC-3'	5'-CTT TAG TAC AGT AGC ATA CTA TC-3'
10	5'-CCG TAA ACT GAA GGG TAT TTG-3'	5'-CCT GTT TGG AAT GAA GAA CAT-3'
11	5'-CAC TGG AAG TGC TTT AAG GC-3'	5'-CTG AAG TTT AGA AAG GGA GAC-3'
12	5'-CAG GCA TCC AGG TAG ACT G-3'	5'-CAC GTA TCT CCT GTG CCA G-3'
13	5'-CAG CAC CAA GGG GCA CGC-3'	5'-CTC ACA CCC CAA AAG GTT AG-3'
14	5'-CGT CTG ATG TAT GCA GGT AC-3'	5'-CCA TCG ACT GAA CCT GGA G-3'
15	5'-GCT GGA GGG ATG TGT TGA TC-3'	5'-GAA CAC TTA AAC TTC CCA ACT G-3'
16	5'-GAT GTT ACG TGG ATC CAA GC-3'	5'-CAA CAT CAC TGG AAG AAA TAC C-3'

### Computational Modeling and Electrostatic Calculations

The coordinates of mammalian AMPK were obtained from the protein data bank (accessation code 2V8Q) [16], [17]. The wild type structure of the regulatory domain of mammalian AMPK and the K485E mutant was built from the existing sidechain using the Mutator plug-in for VMD [18].

To understand the effect of the K485E mutation on the structure of AMPK, the Poisson-Boltzmann equation was solved analytically [19] for both the wild type and the mutant protein. Dielectric constants of 1 and 78.54 were used for the solute and solvent, respectively. A single ion was used as the boundary condition and the charge was discretized using a cubic B-spline interpolation with harmonic average smoothing applied to the surface. The solvent radius was 1.4A, the spline window was 0.3A, the system temperature was taken to be 298.15 K, and 129 grid points were used in all dimensions. Sequence alignments and comparisons were performed using BLAST to assess the conservation of the residues involved in the salt bridge [20].

## Results

Nineteen patients diagnosed with HCM (14 male and 5 female) with a mean age of 31±3 years of age (15 to 46 y/o at diagnosis) were enrolled for clinical evaluation. Clinical data of all patients is listed in Table 2. Maximal left ventricular wall thickness ranged from 16 to 36 mm (24±1 mm), and in 4 patients exceeded 30 mm. Eight patients had evidence of significant left ventricular outflow tract obstruction as evidenced by the presence of a systolic pressure gradient greater than 30 mmHg at rest. Fifteen of the 19 subjects presented with asymmetric hypertrophy. All subjects had normal RA and RV values. Seventeen of 19 subjects had normal or enhanced LV systolic function with ejection fractions of 57% or more (70% ±2%), all of which had normal LV cavity dimensions. We identified 4 cases with bradycardia, and among them was one displaying electrocardiographic manifestations of preexcitation, in association with a markedly enlarged LV with impaired systolic function, AV block, tachyarrhythmia and atrial fibrillation (AF) with rapid ventricular rates.

**Table 2:** Clinical characteristics of patient with hypertrophic cardiomyopathy.

Patient No.	Gender	Age at Dx	Symptom	BP (mmHg)	Family history	Echocardiography						ECG
						LA (mm)	LV (mm)	IVS (mm)	LVPW (mm)	LVEF	LVOT gradient (mmHg)#	
1	M	38	chest tightness, chest pain, palpitation	121/80	Y (HCM)	31	38	17	9	79%	39	N/A
2	M	41	chest tightness, chest pain	120/80	N	33	45	16	11	70%	<30	LV hypertrophy, abnormal repolarization
3	M	31	syncope	95/60	N	40	40	32	9	71%	<30	LV hypertrophy, abnormal repolarization
4	F	37	chest tightness, shortness of breath	109/68	Y (HCM)	43	45	20	12	63%	56	LV hypertrophy, abnormal repolarization
5	M	16	syncope	117/49	N	35	43	20	11	63%	<30	left and right atrial enlarged, RBBB
6	M	34	chest tightness, shortness of breath	92/50	N	28	31	27	9	81%	47	sinus bradycardia, LV hypertrophy, and abnormal repolarization
7	F	46	palpitation	128/68	N	43	48	19	9	59%	<30	sinus bradycardia, abnormal repolarization
8	M	21	chest tightness	120/54	N	36	38	36	24	57%	<30	LV hypertrophy, abnormal repolarization
9	M	39	chest tightness, palpitation	144/90*	Y (HCM)	40	46	27	13	69%	<30	sinus bradycardia, AF, LV hypertrophy, abnormal repolarization
10	M	21	chest tightness	126/70	N	30	38	30	11	61%	32	LV hypertrophy, abnormal repolarization
11	F	19	chest tightness, palpitation, shortness of breath, amaurosis	132/68	N	33	32	21	26	68%	62	LV hypertrophy, abnormal repolarization
12	F	27	palpitation	110/59	N	33	42	17	8	62%	53	abnormal repolarization
13	M	42	chest tightness, chest pain	109/61	N	37	43	28	12	78%	54	LV hypertrophy, abnormal repolarization
14	M	18	chest tightness, palpitation, shortness of breath	120/77	N	44	67**	16	15	30%***	<30	abnormal repolarization
15	M	18	palpitation, shortness of breath	105/55	N	30	37	29	7	79%	<30	LV hypertrophy, abnormal repolarization
16	M	45	chest tightness, palpitation	N/A	Y (HCM)	37	44	16	16	76%	121	abnormal repolarization
17	M	41	shortness of breath	117/61	N	34	38	20	11	80%	39	abnormal repolarization
18	M	15	chest pain, palpitation	155/93*	N	45	65**	31	32	46%***	<30	sinus bradycardia, LV hypertrophy, WPW, AVB, AF, AVRT
19	F	39	chest tightness, chest pain, palpitation	94/51	Y (SCD)	34	36	25	9	66%	138	LV hypertrophy, abnormal repolarization

M = Male; F = Female; Y = Yes; N = No; HCM = Hypertrophic Cardiomyopathy; SCD = Sudden Cardic Death; BP = Blood Pressure; N/A = Not available; LA = Left Atrium; LV = Left Ventricle; IVS = Interventricular Septum; LVPW = Left Ventricular Posterior Wall; LVOT = Left Ventricular Outflow Tract.

* High blood pressure;

** Left ventricular cavity dimension enlarged;

*** LVEF decreased.

# Values at rest.

### Clinical Findings

**Figure 1:**
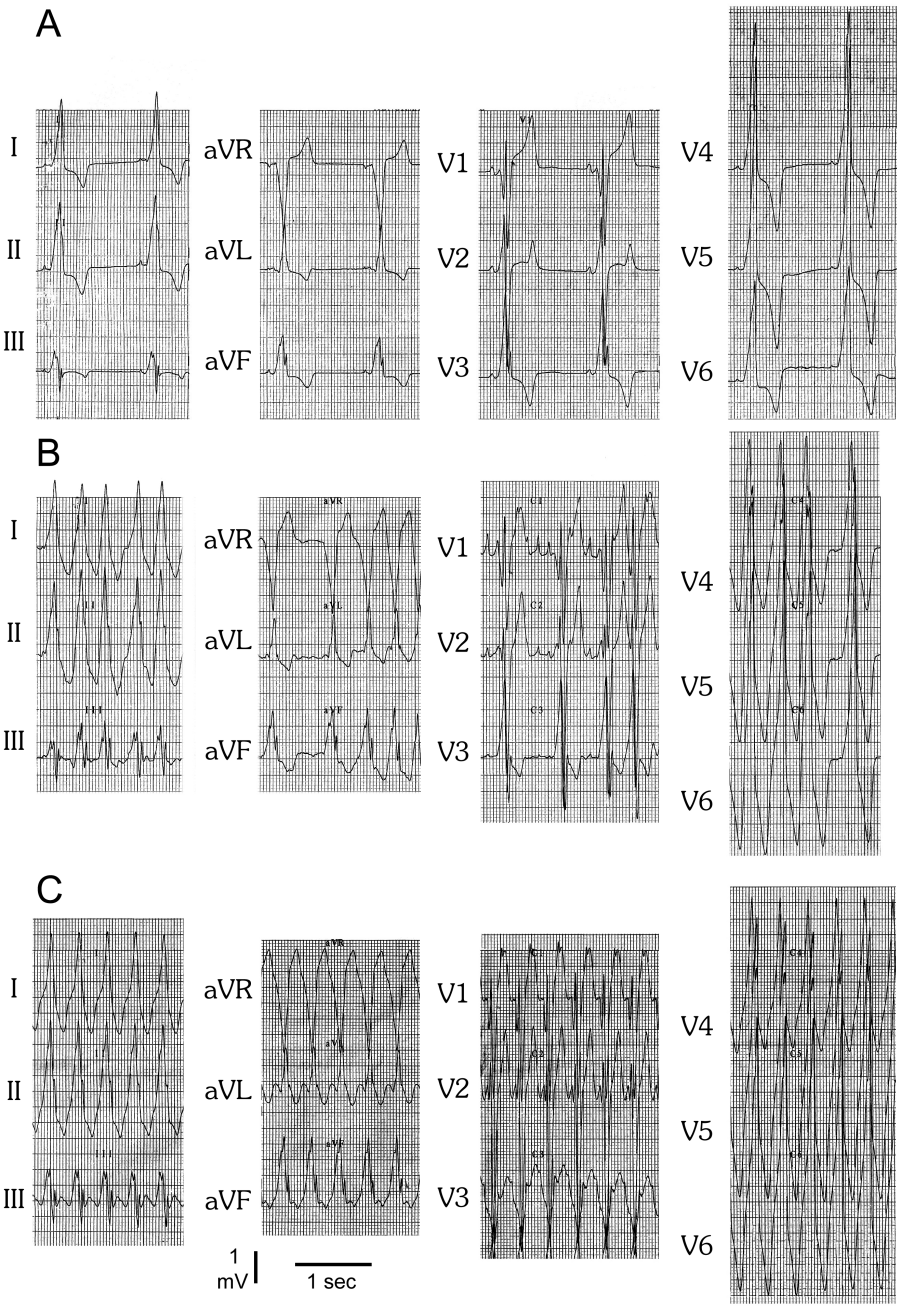
ECGs (25 mm/s, 5 mm/mV) of the proband at age 19. (**A**) Resting ECG shows sinus bradycardia, ventricular preexcitation and significant left ventrical hypertrophy. (**B**) Irregularity of the rhythm, rapid ventricular response, delta waves, and wide, bizarre QRS complexes with beat-to-beat variations in configuration, strongly suggesting the diagnosis of combined AF and WPW. (**C**) Regular wide QRS complex tachycardia, most likely due to antidromic atrioventricular reentry tachycardia (AVRT), suggesting that the accessory connection is capable of sustaining reentry and participating in reciprocating tachycardias.

**Figure 2:**
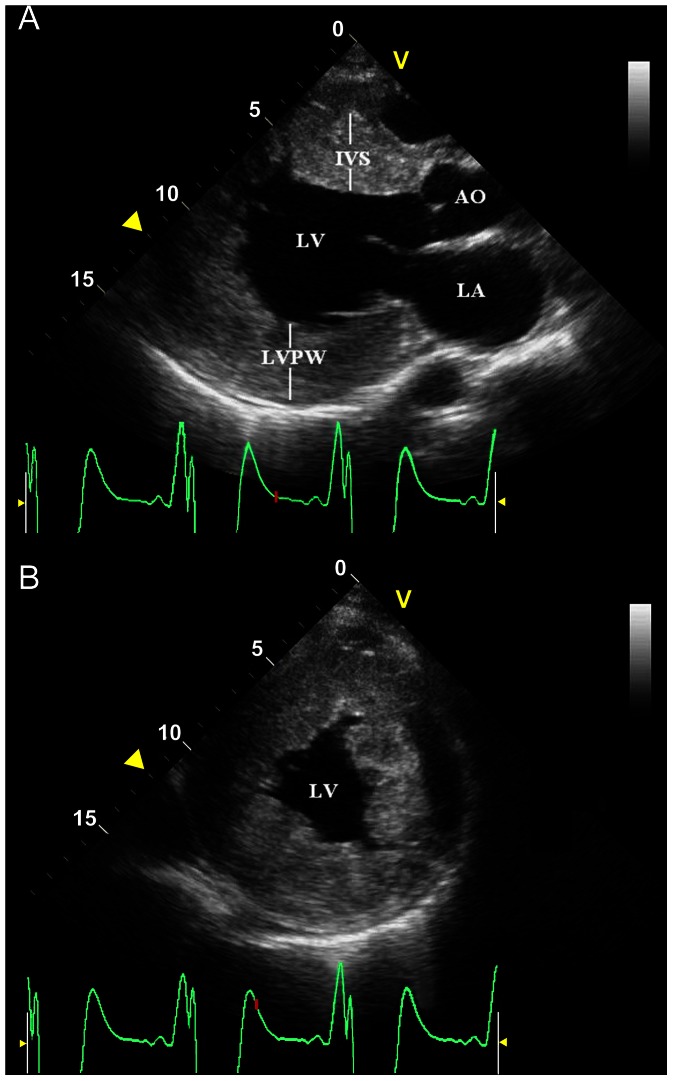
The parasternal long-axis view (A) and short-axis view (B) of an echocardiogram of the proband. It demonstrates severe concentric left ventricular (LV) hypertrophy with enlarged left chambers. AO, aorta; IVS, interventricular septum; LA, left atrium; LVPW: left ventricular posterior wall.

The case is a 19-year-old Chinese male, initially presented with palpitations and chest pain. He was diagnosed with HCM, WPW and hypertension at the age of 15. The resting ECG at admission showed sinus bradycardia (50 bpm), ventricular preexcitation and significant LVH (Figure 1A). He experienced episodes of AF with ventricular rates up to 214 bpm (Figure 1B) and antidromic atrioventricular reentry tachycardia (AVRT) with a rate of 167 bpm (Figure 1C). The QRS morphology during sinus bradycardia, AF and AVRT was similar (Figure 1), suggesting antegrade conduction via the accessory pathway with variable ventricular pre-excitation. Echocardiography revealed marked concentric LVH (septal thickness of 31 mm and posterior wall thickness of 32 mm) without signs of resting left ventricular outflow tract obstruction, an enlarged left atrium (diameter, 45 mm) and left ventricle (end-diastolic diameter, 65 mm), a left ventricular ejection fraction of 46% with mild left ventricular wall diffused hypokinesia and a small pericardial effusion (Figure 2). The right side of the heart was normal in size and function. Serum biomarkers for liver function were normal (ALT 38 U/L, AST 32 U/L, CK 96 U/L).

**Figure 3:**
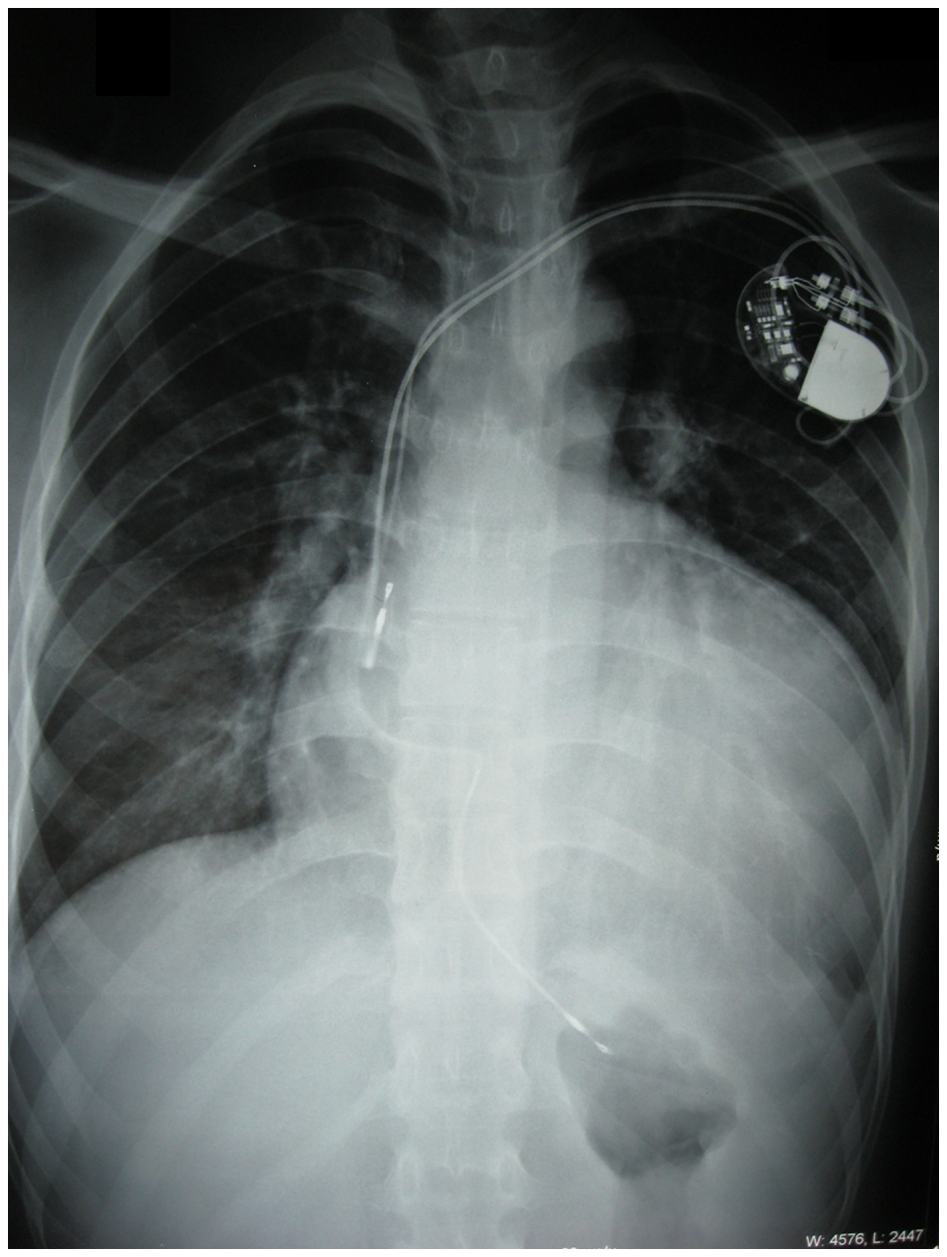
Postero-anterior chest radiograph of the proband after permanent pacemaker implantation. It shows severe cardiomegaly with a cardiothoracic rate of 70.6%.

Electrophysiologic study revealed an atrioventricular accessory pathway in the anteroseptal region. Given an increased risk of progression into ventricular fibrillation and sudden death in patients with preexcited AF, radiofrequency catheter ablation (RFCA) of the bypass was attempted. Radiofrequency energy was delivered at the site with the earliest ventricular activation. Immediately (3 seconds) after radiofrequency energy delivery, the patient experienced transient complete AV block without ventricular escape rhythm. Finally, a permanent dual-chamber pacemaker was implanted. The patient became completely pacemaker dependent. Severe sinoatrial node dysfunction was confirmed by the fact that absence of spontaneous rhythm was recorded on ECG when his pacemaker was temporarily programmed to a rate of 40 bpm. Furthermore, because accessory pathway conduction remained in this patient, a second ablation procedure was performed to eliminate recurrent pre-excitation. An AV accessory pathway at a site close to the originally ablated pathway was detected. RFCA successfully eliminated the pathway conduction. Subsequent echocardiography showed no obvious change of cardiac structure and function compared to his initial presentation. The chest X-ray showed severe cardiac enlargement with a cardiothoracic rate over 70% (Figure 3). Over a 3-year follow-up period, the proband took amiodarone (200 mg daily) and perindopril (4 mg daily) and no recurrences of AF were detected. The patient experienced progressive deterioration of cardiac function with intermittent dyspnea on exertion and orthopnea, which can be alleviated by oral administration of digoxin (0.125 mg daily) and DHCT (25 mg every 12 hours). More recently, he suffered from pulmonary edema and acute congestive heart failure induced by pneumonia, and is awaiting cardiac transplantation.

The proband’s father, 60 y/o, with normal blood pressure and no medical history of cardiovascular diseases, exhibited a resting ECG showing sinus bradycardia (56 bpm) and increased LV voltage. Echocardiography exhibited mildly dilated left atrium (diameter, 38 mm) and LV (end-diastolic diameter, 53 mm) and widening of the proximal ascending aorta (diameter, 42 mm) without cardiac hypertrophy. His mother and brother are asymptomatic and negative for all clinical exams.

### Histopathological Analysis

**Figure 4:**
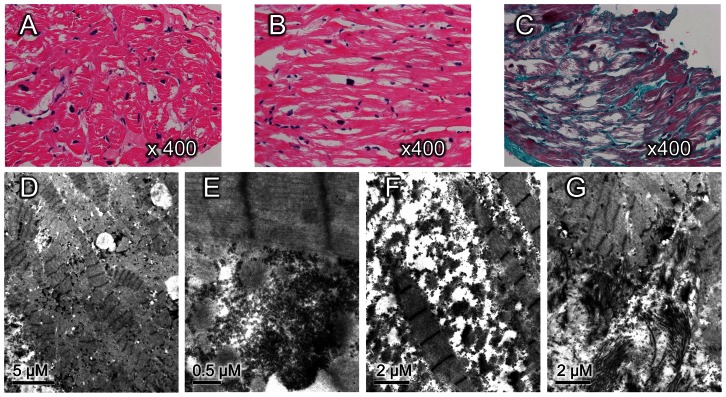
Histopathology of ventricular sections obtained from the proband. (**A**) Cross section of ventricular myocardium shows hypertrophied myocytes with profound intracellular vacuolation and marked variation in the nucleus size (hematoxylin and eosin staining, original magnification × 400). (**B**) Myocardial fibers in longitudinal section shows loss of the normal parallel arrangement, increased side-branching, and frequent side-to-side branches connections (hematoxylin and eosin staining, original magnification × 400). (**C**) Masson’s trichrome staining shows increased interstitial fibrosis with blue color (original magnification × 400). (**D**) Electron microscopy shows angulated myofibrillar with length-heterogeneity sarcomere lengths and excess mitochondria (bar = 5 µm). (**E**) Granular glycogen accumulation in the subsarcolemmal regions of cardiac myocytes (bar = 0.5 µm). (**F**) Myofibrillar dissolution and widening of intermyofibrillar spaces in which scattered glycogen granules and mitochondria are seen (bar = 2 µm). G, Prominent interstitial fibrosis and myofibrillar disarray (bar = 2 µm).

Light microscopy evaluation of the proband’s heart tissue obtained by biopsy revealed marked enlargement of myocytes with profound intracellular vacuolation, pleomorphism of nuclei (Figure 4A), loss of the normal parallel arrangement, frequent side-to-side connections (Figure 4B), and increased interstitial fibrosis (Figure 4C). Electron microscopy confirmed significant loss of myofibrils and myofibrillar disarray (Figure 4D). Markedly increased glycogen content was found in the interfibrillar, perinuclear and subsarcolemmal regions of cardiac myocytes (Figure 4E). There were no obvious mitochondrial morphological changes, but the number of mitochondria increased significantly, and clustered mitochondria showed the characteristic of regional accumulation (Figure 4D). No crystal formation was detected in mitochondria. Electron-lucent droplets of various sizes, suggestive of lipid, were frequently observed. Occasionally, large accumulations of lipofuscin were found in partial cells. In minority cells, there were irregular empty areas between myofibrils with the presence of scattered glycogen and mitochondria, which may be due to dissolution of glycogen (Figure 4F). Interstitial fibrosis was prominent and highly aggregated in partial areas (Figure 4G).

### Molecular Genetics Analysis in *PRKAG2* Gene

**Figure 5:**
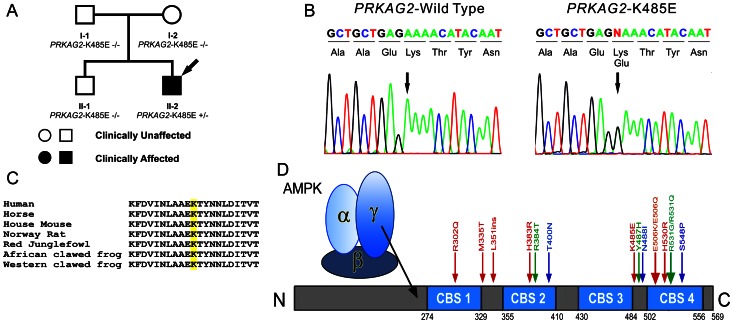
Genetic analysis identified a novel *de novo PRKAG2* mutation. (**A**) Family pedigree of the *PRKAG2* mutant carrier. Arrow denotes proband. (**B**) DNA chromatogram shows a heterozygous A-to-G transition at nucleotide 1453 of *PRKAG2*, predicting a substitution of a glutamic acid for lysine at residue 485 (p.Lys485Glu) of the AMP-activated protein kinase (AMPK) γ-2 subunit (K485E). (**C**) Amino acid alignments show that a lysine at position 485 is highly conserved among species. (**D**) Schematic of AMPK γ-2 subunit and all *PRKAG2* mutations discovered by far. Residue K485 is located at the linker between the third CBS domain and the fourth CBS domain.

Genetic screening of the patient revealed a novel heterozygous mutation consisting of an A-to-G transition at nucleotide 1453 (c.1453A>G) in *PRKAG2* predicting a substitution of a glutamic acid for lysine at residue 485 (p.Lys485Glu, K485E) of the AMPK γ2 subunit (K485E, Figure 5B). This mutation was not found in reference alleles from 215 healthy controls, as well as HGMD database (www.hgmd.cf.ac.uk) and previous publications. This mutation was also absent in all family members, who were all negative for the syndrome (Figure 5A). Paternity testing supported the conclusion that the genetic variation uncovered in the proband was a *de novo* mutation. We also prudently validated this mutation by the same sequencing platform for preventing a false positive result of *de novo* mutation. Alignment of the amino acid sequence of the γ2 subunit proteins showed that lysine at position 485 is highly conserved among species (Figure 5C). Residue K485 is located at the linker between the third and forth CBS domain (Figure 5D). Genetic screening of the remaining 18 patients was all negative. This mutation is predicted to be possibly damaging with a score of 0.820 by polyphen, and to be damaging with a score of 0.04 by SIFT.

### Computational Modeling Prediction

**Figure 6:**
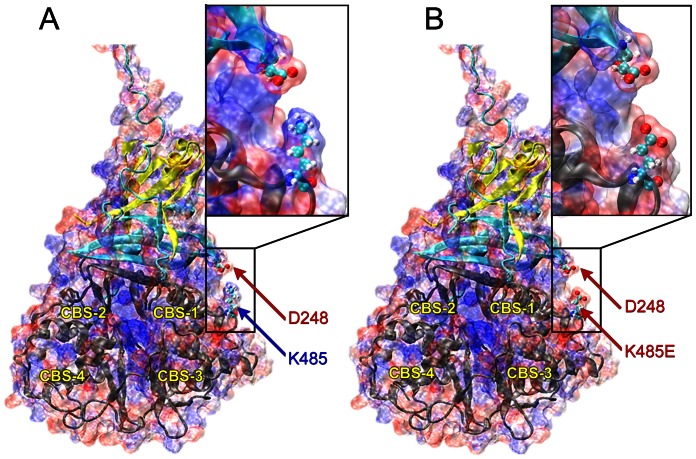
Electrostatic surfaces of (A) WT AMPK and (B) the K485E mutant. Positively- and negatively-charged regions on the surface of the protein are shown in red and blue, respectively. The α, β, and γ subunits of the protein are colored in yellow, blue, and gray cartoons, respectively. The four CBS domains of the protein are also labeled for reference. The D248 and K485 residues are shown in ball-and-stick representation and labeled with arrows and insets provide a close-up look at the effect of the mutation on the salt bridge.

Electrostatic analysis of the mammalian AMPK protein indicates that the lysine (K485) provides a surface of positive charge on the γ subunit, which is positioned to interact strongly with a surface of negative charge on the β subunit around an aspartate residue (D248). Mutation of the lysine to a glutamate results in this positively-charged surface being replaced by a negatively-charged surface, which will repel the negative aspartate (Figure 6). This evidence leads to the conclusion that a salt bridge formed between K485 and D248 plays an important role in the regulatory mechanism of AMPK mediated by the binding of AMP and ATP to the γ subunit.

The lysine residue in the γ subunit is conserved across the AMPK family, but it is not a conserved feature in other families that contain CBS domains (e.g. the chloride channel family and the inosine monophosphate family), indicating that this lysine residue is likely involved in the regulatory interaction between the β and γ subunits. Further evidence for the importance of this salt bridge in regulation of AMPK is provided by the conservation of the aspartate (D248) in the β subunit of the AMPK family. This residue is conserved in all isoforms of the AMPK protein as well as in several members of the SNF1 family, notably Sip1, Sip2, and Gal83.

The separation distance between the lysine and aspartate residues directly affects the strength of the salt bridge and thus would be anticipated to have an important role in mediating the regulation of the protein. Evidence from crystal structures of mammalian AMPK with a variety of ligands bound in the γ subunit [16], [17], pointed to the important role of this salt bridge in the regulatory mechanism. When the activating ligands AMP or ADP are bound to the protein, the salt bridge is formed at a distance of approximately 6 Angstroms; when the inhibitory ligand Mg-ATP is bound, the salt bridge is lost as the distance between the two residues increases to more than 9 Angstroms.

## Discussion

AMPK is a highly conserved heterotrimeric protein comprised of a catalytic α and regulatory β and γ subunits. It is an important energy-sensing enzyme that monitors cellular energy status, maintains energy balance and functions by phosphorylation of key enzymes in lipid metabolism, which leads to decreases in fatty acid synthase and increases in fatty acid oxidation, and by increasing expression and translocation of glucose transporters, which enhances glucose uptake [21]. *PRKAG2* gene, located at 7q36 [22], encodes the AMPK γ2 regulatory subunit, which comprises 4 cystathionine beta-synthase (CBS) domains [23]. Each pair of CBS sequences forms a nucleotide-binding module called the Bateman domain. In humans, three *PRKAG2* transcriptional splice variants, encoding different isoforms, have been identified [22], [24]. Function attributed to the γ2 subunit is to regulate the AMPK activity by binding 2 molecules of either AMP or ATP in the CBS domains [25]. AMPK is activated by AMP and inhibited by ATP, but the homeostasis can be disrupted by disease mutations in *PRKAG2*. AMPK carrying *PRKAG2* mutations are reported to have decreased affinity for ATP, causing inappropriate baseline activation of the enzyme [23].

Since the original discovery by Blair *et al* [2] and Gollob *et al* [3] in 2001, fourteen *PRKAG2* mutations (Figure 5D), including a 3-bp insertion and 13 missense mutations have been identified [7], [23], [26]–[28]. Each mutation has been shown to cosegregate with the disease phenotypes with complete penetrance. In the present study, we identify a novel *de novo PRKAG2* mutation (K485E) between CBS3 and CBS4. This missense mutation predicts substitution of a glutamic acid (E), a negatively charged polar molecule, for lysine (K), a positively charged polar molecule. Calculations of the electrostatic surface of the molecule indicate that K485 is proximal to a negatively charged aspartate residue (D248), forming a putative salt bridge between the β and γ subunits of AMPK. Conservation analysis indicates that both of these residues are conserved not only in the AMPK family, but in many members of the related SNF1 family as well, demonstrating the importance of this salt bridge for the proper functioning of the protein. By comparing the K485-D248 distance in crystal structures with AMP, ADP, and Mg-ATP ligands, we have shown that the salt bridge appears to be broken in the presence of the inactivating ligand Mg-ATP. On the basis of these results, we conclude that the salt bridge between K485-D248 aids in the regulation of the enzyme by mediating communication between the β and γ subunits in a ligand-dependent manner, and K485E will break the bond of these 2 residues. *In silico* prediction tools have previously shown that, all pathogenic missense mutations occur in or very close to the CBS domains, and all benign missense variants occur outside the CBS domain region. The K485E mutation discovered in this study just confirms the predetermination again.

In previous studies involving *PRKAG2* defects, progression to advanced conduction system disease was common by the fourth decade of life [3]. However, our patient became pacemaker dependent at the age of 22 due to severe sinus bradycardia and complete AV block. This may be explained by the fact that the presence of AV block is initially masked by the preexcitation and underlying conduction through the AV node may manifest heart block after the accessory pathway loses its ability for anterograde conduction. However, it is noteworthy that AV node dysfunction in our patient may be iatrogenic because of the manipulation of catheters in the anteroseptal region.

Strikingly, our patient showed early LV dilatation and rapid progression to heart failure. Coincidentally, Blair *et al* [2] also observed in their study that there was a marked propensity towards early cardiac dilatation in patients with *PRKAG2* mutations (Leu351Ins and His383Arg) and the majority of adults either died of heart failure or required cardiac transplantation at an early age. However, *PRKAG2* cardiac syndrome usually progresses slowly with late ventricular dilatation, and is always compatible with long-term survival [12]. The phenotypic disparity may be a result of the specific effects of individual mutations on cellular energy homeostasis and gene transcription by which some may cause a rapid progression toward end-stage heart failure, whereas others do not. In patients with classic HCM, evolution to severe progressive heart failure is uncommon; furthermore, most patients with non-obstructive HCM probably do not develop severe progressive heart failure during their clinical course [29]. The phenotypic heterogeneity therefore may help to distinguish these disorders.

Our histopathological studies revealed increased glycogen content in cardiomyocytes and intracellular vacuolation which highlight the fact that glycogen storage is the pathologic basis of *PRKAG2* syndrome. However, the unexpected characteristics were the presence of myofibrillar disarray and interstitial fibrosis which significantly involved in partial areas.

Both the *PRKAG2* cardiac syndrome and HCM are characterized by cardiac hypertrophy, but myofibrillar disarray is previously considered to be a pathological hallmark of HCM. Our results extend the clinical spectrum of pathological presentation associated with *PRKAG2* defect and suggest that *PRKAG2* related cardiomyopathy can mimic HCM on pathologic examination and fiber disarray is not pathognomonic of typical HCM.

Our patient also had hypertension which was observed in previous reports and speculated to be due to endothelial dysfunction-mediated by AMPK [30]. In addition, although the proband’s father also presented with increased LV voltage on the resting ECG, the echocardiographic assessment did not exhibit LVH and genetic testing for *PRKAG2* was negative. We therefore infer that degeneration with increasing age might be the cause of the father’s cardiac abnormality according to the evidence of nonspecific cardiac changes including sinus bradycardia, mildly dilated LV and widening of the proximal ascending aorta.

Identification of the molecular substrate is important for determining the appropriate strategies for management in patients with unexplained LVH due to the different clinical courses associated with HCM or glycogen storage diseases. Our data demonstrate significant differences in clinical course between patients with unexplained LVH but without electrophysiological abnormalities and the patient with both, including early onset of left ventricular dilatation and rapid progression of heart failure. To date, there is no specific medication for *PRKAG2* cardiac syndrome. Lifelong follow-up of the mutation carrier is necessary because of the high incidence rate of progressive cardiac conduction diseases. Cardiac transplantation might be the optimal option for patients with the last-stage of the *PRKAG2* cardiac syndrome.

### Study Limitations and Conclusion

The study cohort was relatively small and our observations need to be confirmed in studies of larger cohorts. Investigation of the functional consequences of the *PRKAG2-*K485E mutation is not available and would be most welcome in the form of studies involving transgenic mouse models as well as in human stem cell models. Whereas, K485E is already predicted to deleterious by both Polyphen, SIFT, and computer modeling tool.

In summary, we identified a novel *de novo PRKAG2* mutation (K485E) in a young patient with ventricular preexcitation, conduction defect, cardiac hypertrophy and rapid progression of heart failure. The K485E mutant breaks the salt bridge between the β and γ subunits of AMPK and thus affects the function of the protein. Our findings extend our knowledge of the phenotypic and genotypic expression of *PRKAG2* cardiac syndrome, highlighting the fact that excessive glycogen storage is a hallmark of this condition. Although the cardiomyopathic process of *PRKAG2* cardiac syndrome is not caused by primary genetic defects in cardiac structural proteins, this disease also shows fiber orientation disorder similar to that found in HCM, which implies a need for continued morphologic characterization of cardiomyopathy, in conjunction with genetic testing. Preexcitation related to *PRKAG2* mutations can be treated by percutaneous catheter ablation. Clinical features can sometimes provide effective means of distinguishing between these disorders. Patients who present with unexplained LVH, electrophysiological abnormalities, early onset of marked LV dilatation and heart failure without elevations of serum CK and ALT should be suspected of *PRKAG2* cardiac syndrome. Although rare, *PRKAG2* syndrome can present as a sporadic case in which progression of the disease warrants close medical attention.
